# Rhamnazin attenuates Amyloid β-Peptide (1–42) induced spatial memory impairments by modulation of BDNF-ERK signaling pathway

**DOI:** 10.1007/s13205-026-04810-2

**Published:** 2026-05-18

**Authors:** Sajjad Jabbari, Zainul Amiruddin Zakaria, Irwin Rose Alencar de Menezes, Saeed Mohammadi

**Affiliations:** 1 Department of Biology, Faculty of Sciences, Islamic Azad University, Tehran North Branch, North End of Sattari Highway, Simon Bolivar Blvd, Hesarak Ave, Tehran, 14778-93855 Iran; 2 Department of Biomedical Sciences, Faculty of Medicine and Health Sciences, University Malaysia Sabah (UMS), 88400 Kota Kinabalu, Sabah Malaysia; 3 Department of Pharmaceutical Sciences, Faculty of Pharmacy, Airlangga University, Gedung Nanizar Zaman Joenoes Kampus C UNAIR, Jl. Mulyorejo, Mulyorejo, Surabaya, East Java 60115 Indonesia; 4 Laboratory of Pharmacology and Molecular Chemistry, Department of Chemical Biology, Regional University of Cariri (URCA), Rua Coronel Antônio Luis 1161, Pimenta, Crato, Ceará CEP 63105-000 Brazil; 5 Department of Biology, Science and Research Branch, Islamic Azad University, North End of Sattari Highway, Simon Bolivar Blvd, Hesarak Ave, Tehran, 14778-93855 Iran

**Keywords:** Rhamnazin. Memory. Alzheimer. BDNF. Hippocampus. Extracellular signal-regulated MAP kinases

## Abstract

Rhamnazin (Rham), a natural flavonoid, possesses various medicinal benefits including anti-inflammatory, antioxidant, antiangiogenic, and antibacterial activities. Additionally, Rham showed neuroprotective effects when assessed using the chronic stress-induced cognitive impairment assay. In this intriguing investigation, researchers delved into the working memory and spatial reference memory of Rham, utilizing a rat model of Alzheimer’s disease (AD) induced by amyloid β1-42 (Aβ1-42). Administering Aβ1-42 directly into the ventricles led to notable cognitive impairments in behavioral assessments of rats with AD. However, chronic treatment of Rham (30, 60, and 120 mg/kg) once per day during five consecutive days improved the cognitive functions of AD-induced rats in a dose-dependent manner which was not observed following the acute Rham treatment. Concurrently, Rham administration also increased the levels of BDNF and phosphorylated ERK in the hippocampus. Moreover, the cognitive boost triggered by Rham was replicated through the overproduction of BDNF in the hippocampus. However, this effect was thwarted by either the bilateral delivery of lentiviruses expressing BDNF shRNA into the hippocampus or by a targeted injection of an ERK inhibitor. In conclusion, chronic treatment with Rham improves the cognitive deficits in AD-induced rats possibly via the upregulation of BDNF/ERK signaling pathway in hippocampus.

## Introduction

The most common form of dementia is Alzheimer’s disease (AD), which is characterized by a decline in cognitive function, significant memory loss, personality shifts, and abnormal behavior (Borlikova et al. 2013). Aβ is believed to be a key player in the development of Alzheimer’s disease, with evidence suggesting it contributes to cognitive decline, cell death and neuroinflammation (Korczyn and Grinberg 2024). According to the Alzheimer’s Association Report (2024), approximately 6.9 million Americans age 65 and older are living with Alzheimer’s dementia today. This number could grow to 13.8 million by 2060, barring the development of medical breakthroughs to prevent or cure AD. Various attempts have been made to explore on the possible biomarkers involved in the pathogenesis of AD. Of these, the BDNF/ERK signaling activation involvement in the improvement of cognitive functions related to AD have been one of the interested areas to study (Zhang et al. 2015). Gooney et al. (2002) followed by Gao et al. (2022) have demonstrated the involvevement of both BDNF and ERK in neuronal connectivity and neuroplasticity, suggesting their importance as key mediators of synaptic efficacy (Gooney et al. 2002; Gao et al. 2022). As BDNF is critically involved in neuronal survival, synaptic plasticity, and memory, its serum level is altered in AD patients, suggesting a correlation between the low levels of BDNF with AD-related cognitive deficits (Leal et al. 2017). In a fascinating study by Wan and colleagues (2024), researchers uncovered that rats infused with Aβ1–42 into their lateral ventricles exhibited notable deficits in hippocampus related learning and memory (Wan et al. 2024).

Accumulating evidence also has revealed the importance of association between ERK signaling pathway and the higher functions of learning and memory, which include the regulatory role in the process of synaptic plasticity, through the activity of certain nuclear transcription factors (Peng et al. 2010). Interruptions in the activity within ERK pathway have been linked with neurological syndromes such as AD. For examples, proteins such as tau protein and Aβ that produce pathological deposits in the brain during AD are cytosolic targets of ERK (Morley and Farr 2014; Abyadeh et al. 2024). All of these findings strongly implicated the ERK pathway in the formation of AD and can become a hopeful therapeutic target.

Although, huge amount of money and time spent to carry out research towards finding and developing medications for AD (Borlikova et al. 2013), no treatments can stop or reverse its progression, though some may temporarily improve symptoms. A healthy diet, physical activity, and social engagement are generally beneficial in aging, and may help in reducing the risk of cognitive decline and Alzheimer’s. This problem triggered scientists to search for new drug candidates for the treatment of AD. Moreover, anti-dementia drugs treatment also has been reported to further worsen the health conditions of patients, with adverse effects wavering based on individual response and the specific medication (Borlikova et al. 2013; Geldmacher 2024). For examples, side effects frequently associated with the consumption of acetylcholinesterase inhibitors include nausea, and vomiting. As a result of these unwanted effects reported on the current anti-dementia drugs, the emphasis has been shifted towards developing natural product-based treatments for AD (Korczyn and Grinberg 2024). In fact, anti-AD drugs such as galantamine (a cholinesterase inhibitor) is a natural product isolated from plants of the family Galanthus (Chen et al. 2019).

**Fig. 1:**
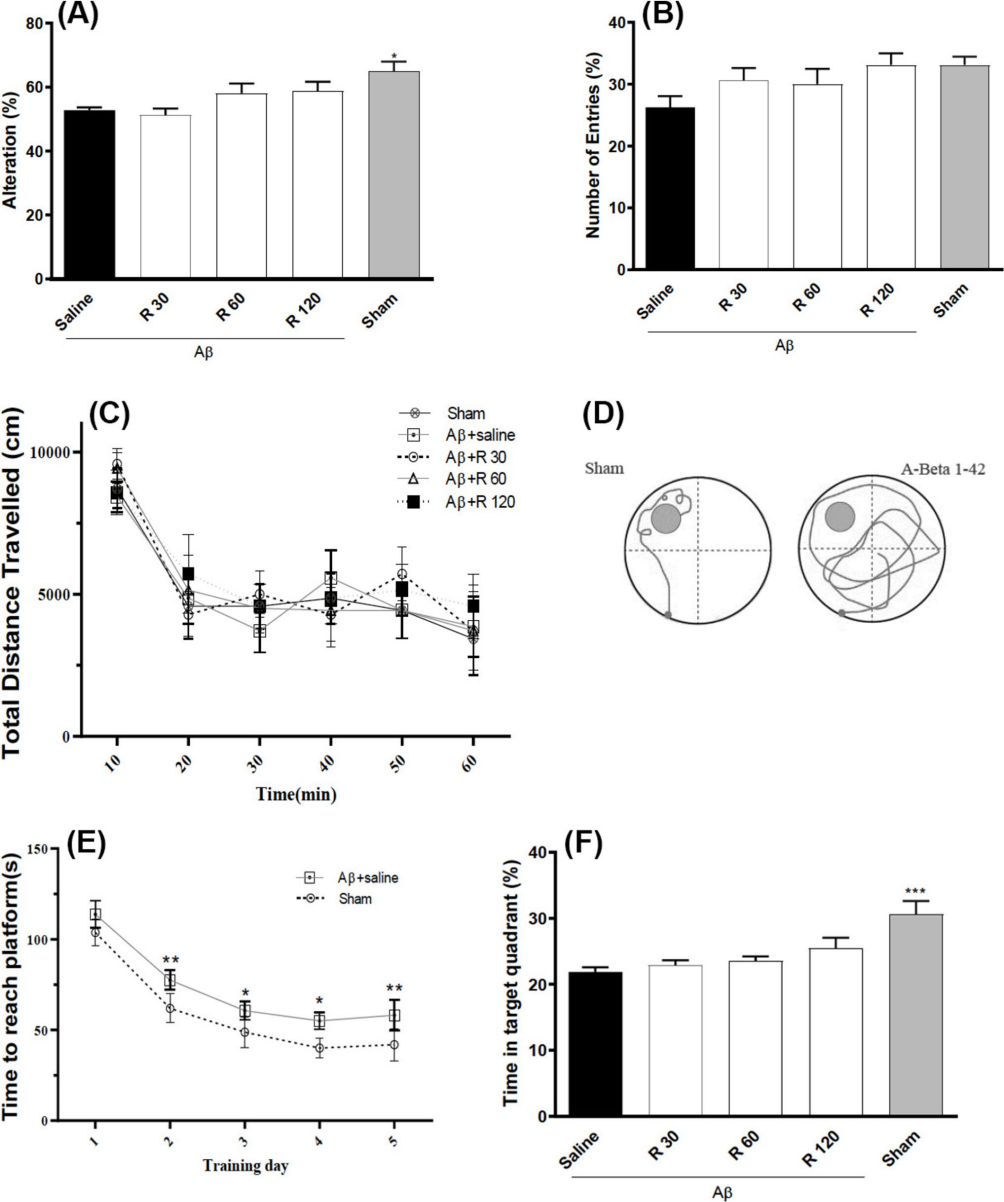
The impact of acute rhamnazin administration on motor skills, spatial memory, and working memory was explored using a rat model of Alzheimer’s disease treated with Aβ1–42. **A** Observations of spontaneous changes in Y maze. **B** The overall number of arm entries recorded in the Y maze. **C** The total distance moved during an OFT assessment. **D** Illustrative swim paths captured during the Morris water maze training sessions. **E** The duration taken to escape into the water maze training sessions. **F** The amount of time spent in the target quadrant during the probe stage. n = 8 in each group. ^*^P < 0.05 and ^**^P < 0.01 and.^***^P < 0.001 compared with Aβ1–42 + saline group. R: Rhamnazin (30, 60, 120 mg/kg)

While the exact ways in which anti-AD treatments work are still shrouded in mystery, the presence of a wide range of beneficial compounds within these plants, such as flavonoids and tannins with each exhibiting diverse pharmacological activities, including anti-amyloidogenic, and anticholinesterase effects, have made them possible candidates for further development of anti-AD drugs (Zhang et al. 2015; Geldmacher 2024). Flavonoids have been shown to possess neuroprotective action and exerted essential protection against neural dysfunction and damages (Calderaro et al. 2022). In addition, Devore et al. (2012) reported that intake of flavanols including rhamnazin (Rham) (Fig. 1S.), which is widely found in medicinal herbs, teas and fruits, could help to improve cognitive performance (Devore et al. 2012). Rham has been proven to exert anti-angiogenesis (Yu et al. 2015), anti-inflammatory (Kim 2016) and neuroprotective (Saeed Mohammadi 2018) activities. Rham was also shown to ameliorate traumatic brain injury by reducing the presence of oxidative stress and apoptosis (Yang et al. 2022). Taking into consideration of the previous reports (Saeed Mohammadi 2018; Mohammadi et al. 2020; Yang et al. 2022) on the beneficial effect of Rham and ability of this amazing flavonoid to transfer across blood–brain barrier ^20^, the present study set out to explore the possible potential of Rham in safeguarding against cognitive deterioration associated with Alzheimer’s disease and to evaluate the potential role of BDNF/ERK signaling pathway in modulating the action of Rham.

## Material and methods

### Drug preparation

Amyloid β1–42 (Aβ1–42) and rhamnazin (30, 60, 120 mg/kg, Compound CID 5320945, molecular formula: C_17_H_14_O_7_) were purchased from Sigma-Aldrich (USA). Aβ1–42 was solubilized in saline (5 mg/mL) and incubated at 37 °C for 72 h to promote aggregation. Wortmannin (Compound CID 312145, molecular formula: C_23_H_24_O_8_) and PD98059 (Compound CID: 4713, C_16_H_13_NO_3_) were used as pathway modulators. All antibodies and reagents were obtained from Abcam and Cell Signaling Technology (USA/UK). shRNA lentiviral particles targeting BDNF and control constructs were sourced from Santa Cruz Biotechnology (Catalog No. NC9792178 USA).

### Animals and housing

Male Wistar rats (255–290 g, 8 weeks old) were housed in groups of 4 per cage under standard 12:12 h light/dark cycle. Ethics approval was granted by the Science & Research Branch, Islamic Azad University (Approval No: 1403–5563), adhering to NIH guidelines (No: 85–23, rev. 1996). Investigators were blinded to treatment groups. Chloral hydrates were used for all anesthesia procedures.

### Intracerebral microinjection and behavioral assessments

**Fig. 2:**
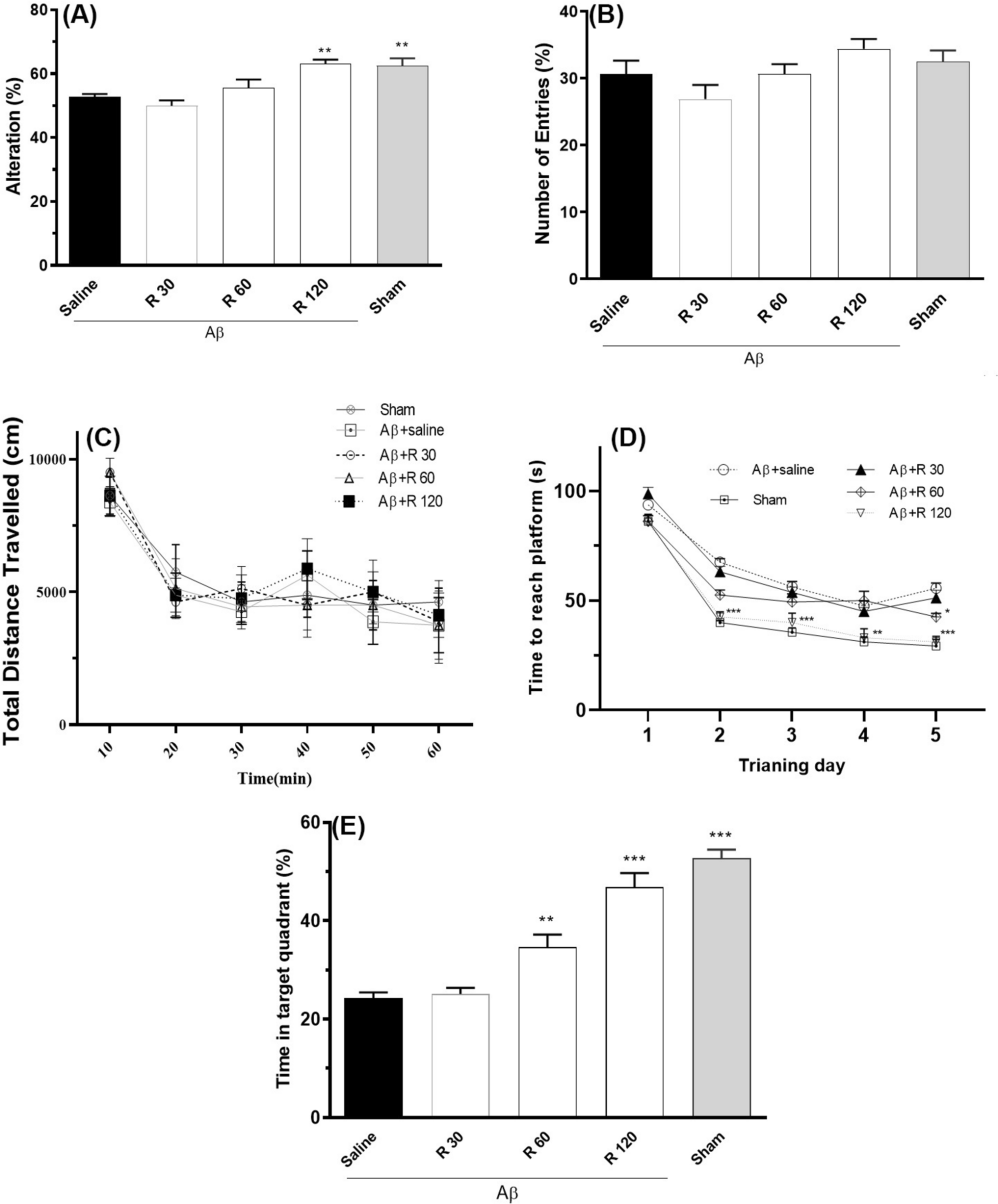
Exploring the impact of long-term rhamnazin therapy on motor skills, spatial memory, and working memory was explored using a rat model of Alzheimer’s-disease treated with Aβ1–42. **A** Observations of spontaneous changes in Y maze. **B** The overall number of arm entries recorded in the Y maze. **C** The total distance moved during an OFT assessment. **D** The duration taken to escape into the water maze training sessions **E**. The amount of time-spent in the target quadrant during the probe stage. ^*^P < 0.05, ^**^P < 0.01 and ^***^P < 0.001 v.s. Aβ1–42 + saline. n = 8 each group. R: Rhamnazin (30, 60, 120 mg/kg). Aβ: Amyloid beta (1–42)

**Fig. 3:**
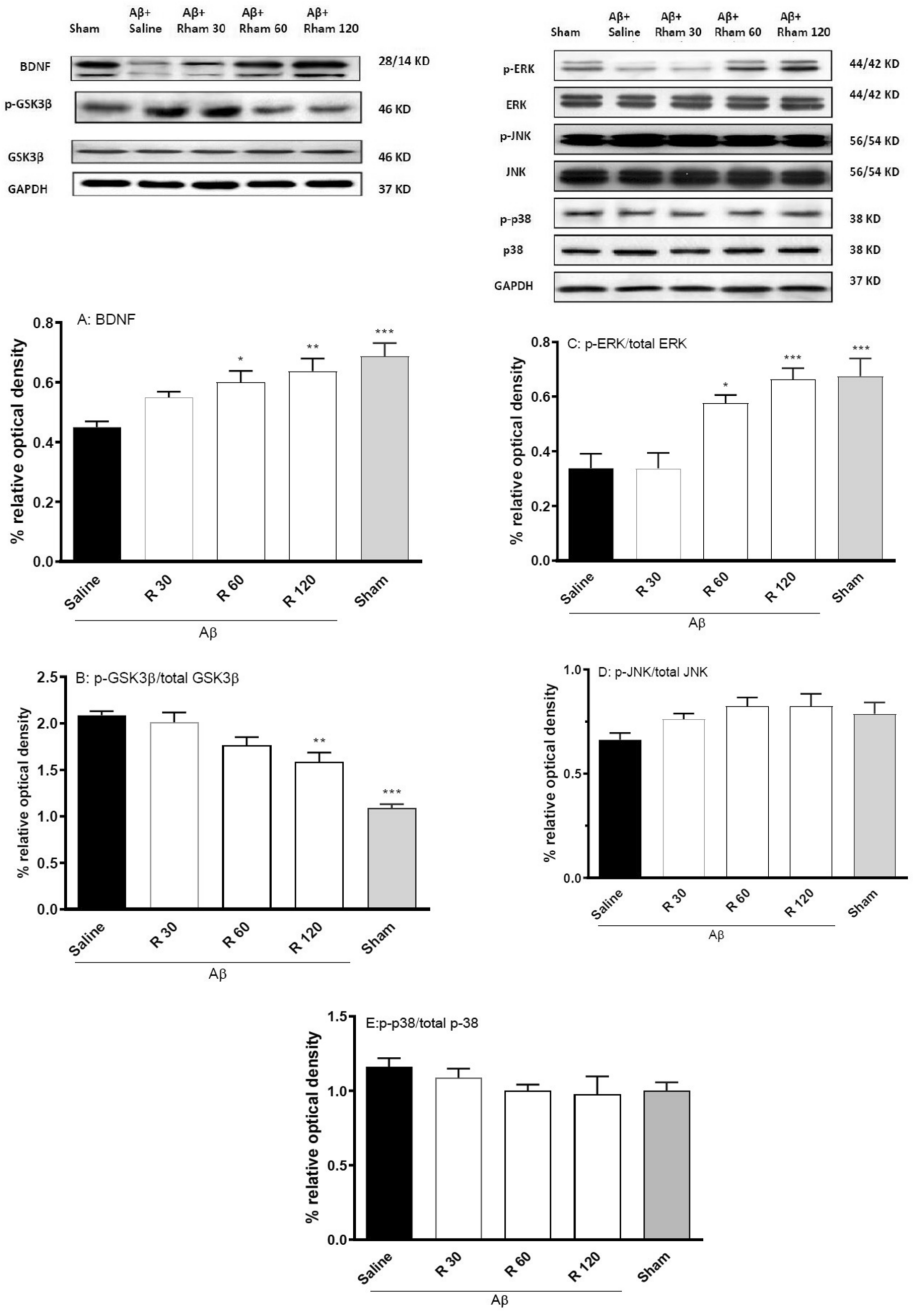
The impact of long-term rhamnazin therapy on BDNF levels and the phosphorylation of MAPK and GSK3β in hippocampus was examined. To ensure accuracy, GAPDH served as the control. The results for protein phosphorylation were presented as the ratio of phosphorylated protein compared to total protein. ^*^P < 0.05 and ^**^P < 0.01 and.^***^P < 0.001 compared with Aβ1–42 + saline. R: Rhamnazin (30, 60, 120 mg/kg). Aβ: Amyloid beta (1–42). Glycogen synthase kinase-3 (GSK3). C-Jun N-terminal kinase (JNK). Extracellular signal regulated kinase (ERK). Glyceraldehyde 3 phosphate dehydrogenase (GAPDH)

Aβ1–42 was administered via bilateral intracerebroventricular (i.c.v.) injection (2.0 μL per side, AP: −0.8 mm; ML: ± 1.4 mm; DV: −4.0 mm). Stereotaxic surgery was performed under anesthesia using chloral hydrate (35 mg/kg, i.p.). Cannulae were fixed with dental cement. For intrahippocampal infusions (targeting dentate gyrus), guide cannulas (30G) were placed (AP: −3.8 mm; ML: ± 2.2 mm; DV: −2.7 mm), and solutions (BDNF, wortmannin, PD98059, or shRNA particles) were infused bilaterally (1.0 μL/side over 5 min – Fig. 2S). Sham groups received saline injections. Microinjections were conducted 30 min prior to behavioral testing. The animals was evaluated by Y-Maze Test, Open Field Test (OFT) and Morris Water Maze (MWM). The experimental protocols for both acute and chronic administration of rhamnazin are detailed in Fig. 3S.

### Western blotting

Hippocampal tissues were homogenized in RIPA buffer, centrifuged, and protein quantified using BCA assay. Proteins were separated on SDS-PAGE, transferred to PVDF membranes, and probed with specific primary antibodies (1:1000). Detection was performed via HRP-conjugated secondary antibodies and ECL substrate. GAPDH served as loading control.

### Acetylcholine quantification

Hippocampal homogenates were processed in sodium phosphate buffer (pH 7.4) and analyzed for ACh using the Amplex Red ACh/AChE Assay Kit (Invitrogen).

### Statistical analysis

Data was analyzed using one- or two-way ANOVA with Bonferroni post hoc tests. One-sample t-tests were used for probe trial analyses. Western blot data were analyzed via densitometry; results were expressed as phosphorylated/total protein ratios. Significance was set at *p* < 0.05.

## Results

### Effect of acute treatment with Rham in a rat model of Alzheimer’s disease

The results showed in a Y-maze test, Aβ-infused rats exhibited a significant reduction in spontaneous alternation relative to sham controls (P < 0.05), and single intraperitoneal doses of rhamnazin (30, 60, or 120 mg/kg) failed to restore alternation rates (all P > 0.98), while total arm entries remained unchanged across groups (one-way ANOVA: F(4,35) = 2.129, P = 0.097 Figs. 1A and 1B). In the open-field test, the total distance traveled declined over time (two-way repeated-measures ANOVA: F(5,210) = 157.4, P < 0.0001) but did not differ by treatment (F(4,210) = 1.686, P = 0.1475 Figs. 1C and 1D), indicating intact locomotor activity. During Morris water-maze training, Aβ-treated rats showed prolonged escape latencies versus sham from days 2–5 (P < 0.05) despite a general acquisition effect over days 1–5 (F(4,28) = 187.6, P < 0.0001 Fig. 1E) and spent significantly less time in the target quadrant on probe day (one-way ANOVA: F(4,35) = 7.560, P < 0.001 Fig. 1F), with no improvement conferred by any rhamnazin dose. These findings demonstrate that, although rhamnazin does not impair locomotion, it fails to ameliorate Aβ-induced deficits in working memory and spatial learning.

### Long-term treatment with Rham boosts cognitive function and elevates the levels of ERK and BDNF in the hippocampus

In this study, chronic administration of rhamnazin (30, 60, and 120 mg/kg, i.p., once daily for five days) produced a dose-dependent improvement in working memory without affecting general locomotion. In the Y-maze, spontaneous alternation was significantly enhanced in the 120 mg/kg group versus Aβ + saline [F (4,35) = 9.890, P < 0.0001; Fig. 2A] (P < 0.01), while total arm entries remained unchanged [F (4,35) = 2.511, P = 0.0593; Fig. 2B], indicating preserved activity levels. In the open-field test, the total distance traveled varied over time [F (5, 180) = 131.7, P < 0.001], but did not differ between treatments [F (4, 180) = 1.180, P = 0.321], confirming that rhamnazin does not induce locomotor deficits (Fig. 2C).

In the Morris water maze, rats receiving 60 and 120 mg/kg rhamnazin exhibited markedly reduced escape latencies over consecutive days [F_treatment_ (F (4, 175) = 51.83, P < 0.001; F_time_ (4, 175) = 267.3, P < 0.001; Fig. 2D], and in probe trials spent significantly more time in the target quadrant than Aβ + saline controls [F (4, 35) = 40.09, P < 0.001; Fig. 2E]. These findings demonstrate that rhamnazin ameliorates Aβ-induced spatial memory impairment in this model of Alzheimer’s disease.

At the molecular level, five-day treatment with rhamnazin modulated key hippocampal signaling pathways GSK3β, BDNF, JNK, ERK, and p38, along with their activated or phosphorylated versions [Fig. 3 A-E]. BDNF expression was significantly upregulated in the 60 and 120 mg/kg groups (P < 0.05 and P < 0.01) and in sham controls (P < 0.001) versus Aβ + saline. High-dose rhamnazin (120 mg/kg) also reduced GSK3β phosphorylation (P < 0.001) and increased ERK activation (P < 0.05 for 60 mg/kg; P < 0.001 for 120 mg/kg), whereas JNK and p38 phosphorylation remained unchanged. Together, these results identify rhamnazin as a promising modulator of synaptic plasticity and memory through BDNF upregulation, GSK3β inhibition, and ERK pathway activation.

### In the hippocampus, BDNF plays a pivotal role in enhancement of cognition by chronic rhamnazin

In this experiment, rats were treated with chronic rhamnazin (120 mg/kg, i.p.) or saline. Then, a 1.0 μg/side dose of BDNF or a lentiviral-BDNF concoction was carefully microinjected into both sides of the hippocampus precisely 30 min next to the rhamnazin administration. The final analysis included a diverse cast of animal subjects as follows: Aβ + saline, sham, Aβ + BDNF, Aβ + Rham, and the Aβ + Rham + shBDNF group.

**Fig. 4:**
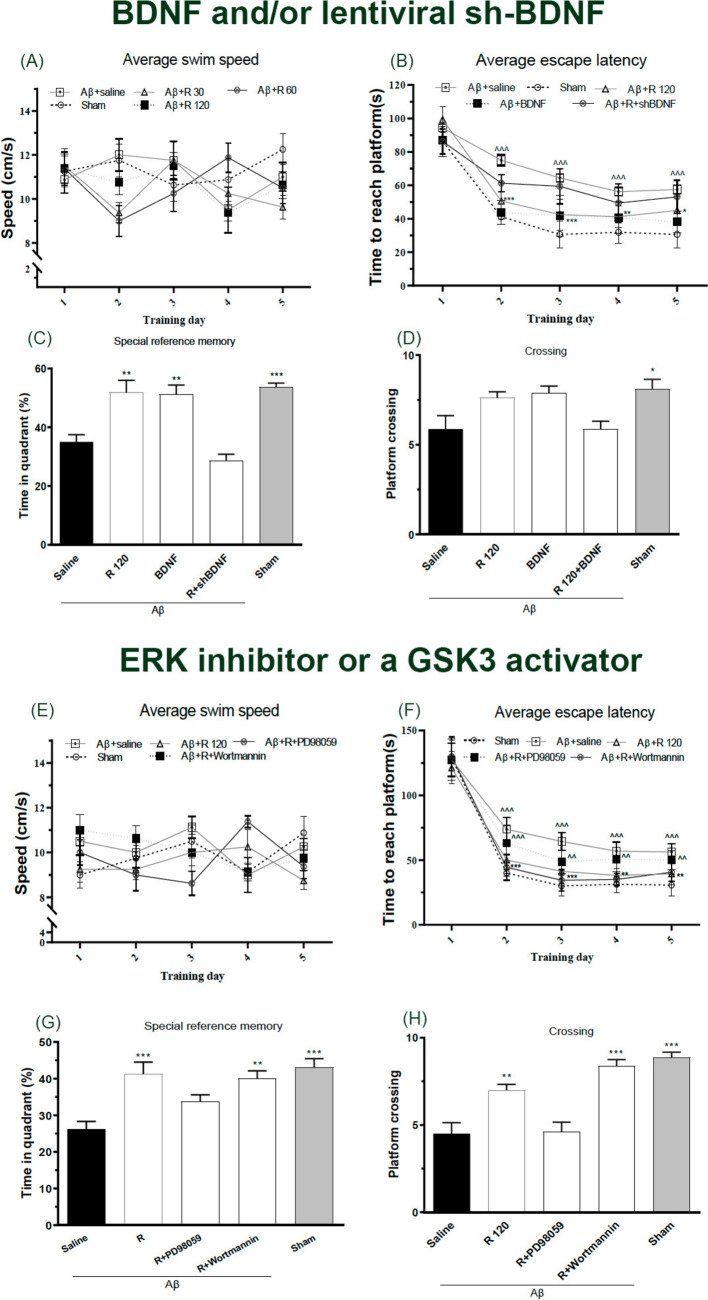
Exploring the impact of injecting BDNF and/or lentiviral sh-BDNF; ERK inhibitor PD98059 or a GSK3 activator wortmannin directly into the hippocampus on spatial learning abilities in an animal model of Alzheimer’s disease. **A, E** Observing the swim speed throughout each training session. **B, F** Measuring the escape latency during the water maze training sessions. **C, G** Tracking the duration spent in the target quadrant and **D, H** counting the number of times the rats crossed the platform during the probe stage. n = 8/group. ^^^^ P < 0.01 and ^^^^^ P < 0.001 vs sham;^*^P < 0.05, ^**^P < 0.01, ^***^P < 0.0001 compared with Aβ1–42 + saline. R: Rhamnazin (30, 60, 120 mg/kg). Aβ: Amyloid beta (1–42). BDNF: Brain derived neurotrophic factor

Throughout the entire water-maze training, the swimming speed of the rats remained consistent, showing no signs of deficits in locomotors activity [Ftreatment (4,175) = 1.140, P = 0.339; F time (4,175) = 1.398, P = 0.236] (Fig. 4A). However, a two-way-ANOVA unveiled a striking impact of the drug treatments [F treatment (4,175) = 55.81, P < 0.001] and time [F time (4,175) = 220.1, P < 0.001] on rat escape latency (Fig. 4B). During the probe trial, time spent in the target quadrant was notably greater for the Aβ + BDNF, Aβ + Rham, and sham groups compared to Aβ + saline (P < 0.01, 0.01, and 0.001, respectively) (Fig. 4C). Interestingly, there were no significant differences in the number of times on the platform was crossed among the Aβ + Rham, Aβ + saline, Aβ + Rham + shBDNF, and Aβ + BDNF (Fig. 4D).

### ERK inhibitors blocked the cognitive improvement brought by rhamnazin, while the GSK3 activator didn’t have the same effect

Given that long-term rhamnazin influences the phosphorylation of both GSK3β and ERK in the hippocampus, we set out to determine which of these elements is more pivotal in enhancing spatial memory linked to the hippocampus. To do this, we administered the GSK3 activator (wortmannin, 100 μM) or the ERK inhibitor (PD98059, 20 μM) Swimming speeds were unchanged across all groups (F treatment(4,175) = 0.51, P = 0.725; F time(4,175) = 0.217, P = 0.928) (Fig. 4E), but rhamnazin significantly reduced escape latencies (F treatment(4,175) = 38.79, P < 0.001; F time(4,175) = 568.9, P < 0.001; interaction F(16,175) = 3.21, P < 0.001) (Fig. 4F). In probe trials, both rhamnazin alone and rhamnazin + wortmannin strongly increased time in the target quadrant (P < 0.001 and P < 0.01, respectively) and platform crossings (P < 0.001 and P < 0.01) versus Aβ + saline, whereas PD98059 completely abolished these benefits (Fig. 4G, H). Thus, ERK—but not GSK3β—phosphorylation is critical for rhamnazin’s enhancement of hippocampal-dependent spatial memory.

### The impact of Rham on the alterations in ACh levels induced by Aβ (1–42)

**Fig. 5:**
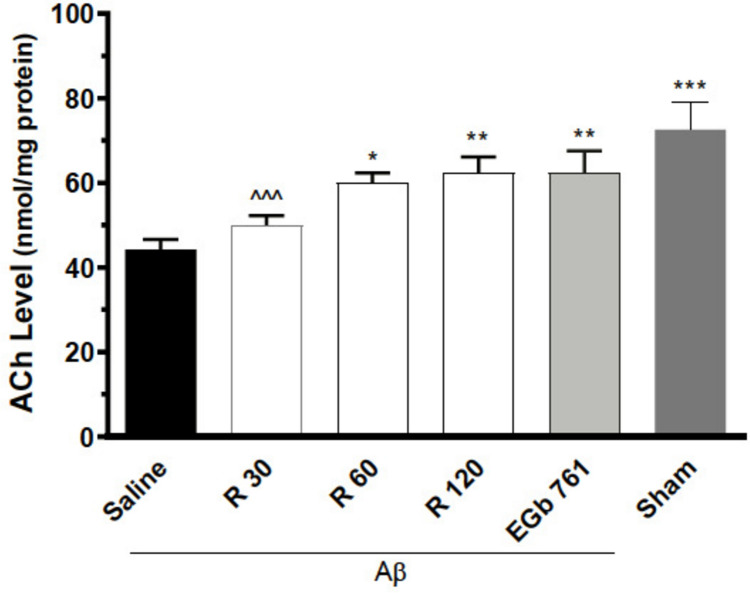
The impact of rhamnazin on fluctuations in acetylcholine (ACh) levels. n = 8/group. ^*^ P < 0.05, ^**^ P < 0.01, ^***^ P < 0.001 compared with Aβ1–42 + saline. ^^ P < 0.01 and ^^^ P < 0.001 vs sham. R: Rhamnazin (30, 60, 120 mg/kg). EGb761: Extract of *Ginkgo biloba* (Tebonin)

A one way-ANOVA revealed notable variations in the ACh levels within the hippocampus across different groups, with a significant statistical result [F (5, 42) = 10.10, P < 0.0001]. There was a remarkable increase in ACh-levels when treated with Rham (60 and 120 mg) and EGb761, compared to the Aβ (1–42) group (Fig. 5).

## Discussion

Studies have shown that Aβ1–42 infusion into the lateral ventricles of rat models is correlated with learning and memory impairments, as evaluated by tasks such as the Y-maze and novel object recognition tests. These impairments are linked to heightened Aβ levels in the brain, which play a pivotal role in the cognitive diminish experienced by individuals with AD. Injecting Aβ1–42 directly into the brains of rodents has been shown to trigger significant memory impairments, mirroring the cognitive deterioration associated with AD (Tamagno and Guglielmotto 2018). In these models, we can evaluate cognitive challenges through a range of engaging behavioral tasks, such as the spontaneous alternation in the Y-maze and the intriguing novel object recognition tests (Miedel et al. 2017). Furthermore, previous studies have revealed that rats exposed to Aβ display significant impairments in spatial learning and memory, particularly in tasks that rely on the hippocampus, affecting both their long term and short term abilities (Wang et al. 2017). The Morris water maze stands out as a popular method for assessing how well the hippocampus handles spatial learning and memory. Research has shown that this process is closely associated with a notable drop in levels of synaptophysin within the hippocampus, along with a disturbance in the expression of BDNF or brain derived neurotrophic factor (Wan et al. 2024).

In our investigation, we observed a similar effect in the control group, showing decreased BDNF levels in the hippocampus. However, repeated administration of rhamnazin, offering a remarkable shield for the cognitive abilities of rats treated with Aβ. Our findings revealed that Aβ treatment led to considerable struggles in learning and memory, particularly evident in their water maze trials. However, the introduction of rhamnazin effectively restoring their cognitive impairments. In the open field test, the rats showed no significant differences in their movement patterns across the groups, suggesting that the memory issues they experienced weren’t associated with changes in locomotion. Instead, these memory challenges were connected to heightened levels of pERK and BDNF in the hippocampus. Interestingly, when BDNF was overproduced in hippocampus of rats injected with Aβ, it replicated the cognitive benefits seen with rhamnazin, but this effect could be thwarted by inhibitors of ERK. Thus, the cognitive benefits of rhamnazin in AD related conditions are primarily mediated through the activation of BDNF/ERK signaling. The neurotrophin BDNF and extracellular signal regulated kinases (ERK) play diverse roles in overseeing the architecture and operations of neurons within both the maturing and fully formed central nervous system.

They have emerged as critical signaling molecules for the development of the nervous system, as well as for impaired nervous systems and multiple diseases, including AD (Nagahara et al. 2009; Chen et al. 2019). As crucial mediators of synaptic efficacy, both BDNF and ERK play a crucial role in the intricate of neuronal connections and ability of the brain to adapt and change (Gooney et al. 2002; Gao et al. 2022). Research reveals that rats infused with Aβ experienced notable deficits in memory linked to the hippocampus. Interestingly, the introduction of BDNF directly into the hippocampus seemed to reverse the alterations brought on by Aβ1–42, restoring cognitive function (Zhang et al. 2015). Curiously enough, even though directly introducing BDNF didn’t seem to improve the decline in learning and memory that comes with age, rodent genetically engineered to produce excess BDNF actually exhibited deficits in learning (Fischer et al. 1994). Based on these findings, it is possible that BDNF induced by rhamnazin specifically targets the deficits caused by Aβ, rather than generally affecting synaptic strength (Lu et al. 2014; Leal et al. 2017). A wealth of research suggests that the natural signaling of BDNF/TrkB is intricately involved in the gene regulation that underpins synaptic plasticity and the memory and learning functions of the hippocampus, all through the lens of ERK activation. Yet, the role of ERK in the development of Alzheimer’s disease is still shrouded in mystery. Interestingly, both excessive and insufficient ERK activation are pivotal in memory processes and could influence the pathways of the disease, as ongoing ERK activation might lead to NMDA related excite toxicity (Ivanov et al. 2006). For instance, the active form of ERK acts as a tau kinase, showing increased levels during the initial phases of neurofibrillary degeneration in the neurons projecting located in the trans entorhinal area (Engin and Engin 2021). Interestingly, in the later phases of the disease, the activation of ERK takes a backseat when compared to both the early stages and healthy controls, particularly within the neuronal cell bodies and the twisted neuritis (Bennison et al. 2020). These findings suggest a fascinating pattern: as ERK activation unfolds in stages, it eventually leads to a decline in levels of ERK activation. Our research indicates that a dip in ERK activation within the hippocampus correlates with cognitive challenges linked to Aβ. This points to the idea that multiple doses of rhamnazin may invigorate ERK signaling. These insights consistent beautifully with earlier investigations into the effects of flavonoids (Pavlova et al. 2016; Calderaro et al. 2022).

Rhamnazin appears to restore the memory and learning skills dulled by Aβ in an Alzheimer’s disease model. It does this by kicking BDNF/ERK/CREB signaling pathway into the hippocampus. Literature data has also unveiled that the Akt/GSK3β signaling pathway plays a crucial role in the positive impact of flavonoids on cognitive challenges brought on by Aβ (Long et al. 2021). GSK3, a serine-threonine kinase, is like a conductor orchestrating a symphony of intracellular signaling pathways within the central nervous system. It takes on the role of a critical gatekeeper, dampening the processes involved in memory formation and cognitive function. Furthermore, GSK3 interacts with several components of amyloid plaques, contributing to the pathogenesis of Alzheimer’s disease (Takashima 2006). AKT, as an important protein kinase, has the remarkable ability to tag and deactivate GSK3 through phosphorylation (Hur and Zhou 2010). Exposure to Aβ could ramp up the GSK3 driven blockade of CREB phosphorylation, which in turn might result in a dip in expression of BDNF (Hur and Zhou 2010).

In our latest investigation, we discovered that infusing ERK inhibitors directly into the hippocampus halted the cognitive boosts brought on by rhamnazin in rats treated with Aβ. Interestingly, a GSK3 activator had no such effect. These findings indicate that ERK is a key player in enhancing spatial memory linked to the hippocampus, while GSK3 seems to take a backseat in this particular experimental scenario. Our findings revealed that Aβ1–42 wreaked havoc on the hippocampal cholinergic system leading to a notable drop in ACh levels. However, the introduction of rhamnazin came to the rescue, significantly mitigating the damage caused by Aβ1–42 to both spatial memory and the cholinergic system. Usually, when presynaptic ACh levels take a hit, one might anticipate a compensatory boost in muscarinic receptors at the postsynaptic sites (Tobin 2024). In a surprising twist, rats treated with Aβ1–42 experienced a drop in ACh levels. This decline could be linked to the neurotoxic effects of Aβ, which seem to disrupt the delicate balance of both pre-synaptic cholinergic nerve terminals and the post synaptic that showcase muscarinic receptors in hippocampus (García-Ayllón et al. 2010). We turned to EGb761, commonly known as Tebonin, as our benchmark medication. This is because its flavonoid elements are thought to play a significant role in boosting memory in individuals with Alzheimer’s disease patients and older rats, partly by enhancing the activity of acetylcholine (Xia et al. 2024; Chen et al. 2024). Similar to EGb761, rhamnazin administration increased ACh levels in the hippocampus of Aβ-induced Alzheimer’s rats. The inhibition of ACh degradation is currently one of the most well recognized therapeutic targets for developing cognitive enhancers.

There is a growing dissatisfaction with a conceptualization of raised amyloid-β and hyper-phosphorylated tau levels in driving the complexity of pathophysiological changes occurring in dementia (Anderson 2023). However, given the ubiquitous benefits of rhamnazin across of host of diverse preclinical medical models in different organs and tissues (Wang et al. 2023), it would seem clear that it is acting on core cellular processes. Data across different organs and tissues shows rhamnazin to modulate signal transducer and activator of transcription 3 (STAT3) (Zhong et al. 2025) and nuclear factor kappa-light-chain-enhancer of activated B cells (NF-κB). Recent work show STAT3 and NF-κB to interact to modulate the induction or suppression of the endogenous melatonergic pathway across diverse human cells (Córdoba-Moreno et al. 2024). As melatonin and its immediate precursor, N-acetylserotonin, up regulate BDNF in the hippocampus, as well as suppressing amyloid-β and hyper-phosphorylated tau induction and toxicity (Anderson 2023), it will be important to clarify whether rhamnazin ubiquitous effects are mediated via the up regulation of the local melatonergic pathway. It will also be important to clarify whether rhamnazin benefits again injected ventricular amyloid-β arise from an increased activation of the glymphatic system, which may also be up regulated by melatonin (Chen et al. 2025).

## Conclusions

To put it succinctly, our findings indicate that rhamnazin effectively shields against memory loss triggered by Aβ1–42 in a way that depends on the dosage. The heightened presence of hippocampus BDNF mirrors the positive impact of rhamnazin on cognitive deterioration in the Alzheimer’s model. Additionally, the ERK signaling pathway, rather than GSK signaling, that is crucial for rhamnazin’s ability to protective against memory challenges caused by Aβ. Our research could shed light on the mechanisms underlying rhamnazin’s protective effects against Aβ-induced cognitive dysfunctions and demonstrates its potential as a candidate for anti-AD therapy.

## Data Availability

Raw data are available upon request to the corresponding author.
